# Establishment of Optimal Drug Delivery System and Evaluation of Utilization of Hydrogel Contact Lens According to the Addition Method of Tretinoin and Bovine Serum Albumin

**DOI:** 10.3390/polym17020159

**Published:** 2025-01-10

**Authors:** Hye-In Park, A-Young Sung

**Affiliations:** Department of Optometry & Vision Science, Daegu Catholic University, Gyeongsan 38430, Republic of Korea; gpdls4731@naver.com

**Keywords:** albumin nanoparticle, hydrogel network, drug delivery, functional materials, hydrogel lens

## Abstract

This study aims to build an optimal drug delivery system by manufacturing and evaluating a hydrogel contact lens using Tretinoin (ATRA) and protein nanoparticles to improve the drug delivery system as an ophthalmic medical contact lens. To evaluate the optical and physical properties of the manufactured lens, the spectral transmittance, refractive index, water content, contact angle, AFM, tensile strength, drug delivery, and antibacterial properties were analyzed. The contact lens was manufactured to contain ATRA and bovine serum albumin (BSA) in different ways, and the results confirmed that A, B, and C each had different physical properties. In particular, for Sample A, using the soak and release method and using ATRA solution in the contact lens with BSA added, the wettability was 55.94°, the tensile strength was 0.1491 kgf/mm^2^, and drug delivery released 130.35 μm over 336 h, which was found to be superior to samples B and C. Therefore, the three hydrogel contact lenses compared in this study according to the addition method of ATRA and BSA can be used in various ways to build an optimal drug delivery system that is very useful as an ophthalmic medical lens.

## 1. Introduction

Hydrogels have a hydrophilic three-dimensional polymer network structure crosslinked by covalent bonds and can absorb large amounts of water and biological fluids [1,2]. The covalent bonding community of gels is characterized by different gel compositions, structures, strengths, and crosslinking technology due to the use of crosslinked polymers [1]. In addition, they have excellent biocompatibility and are widely used in various biomedical and environmental applications such as contact lenses, tissue engineering, molecular implants, medical patches, and drug delivery [2,3]. Hydrogel contact lenses are mesh-structured polymers that form a stable three-dimensional network and have excellent wearability, water content, and drug delivery [4]. They are characterized by the easy control of physical and chemical properties, so if a lens contains molecular chains and nanoparticles with high physical adsorption, its mechanical properties can be easily improved [5]. Therefore, these lenses are used not only for refractive correction and cosmetic purposes, but also for treating eye diseases and diagnosing ophthalmology [6]. The drug delivery system refers to a method of administering and delivering drugs, and it is important to improve the therapeutic effect and safety, target delivery, and drug delivery control [7]. Nanomaterials are widely used as carriers for drug delivery because they are rapidly soluble in the bloodstream due to their small particle size and can reach the target. In addition, they may be designed to selectively target cells and have specific surface properties [8,9]. Gugleva V, Velichka A (2023) reported a 1.5-fold improvement in fibroblast absorption in nanoparticle-encapsulated cargo, and that drug concentrations in the cornea and conjunctiva of rabbits were about 6.7 and 1.3 times higher, respectively, compared to free drugs [10]. Among them, protein nanoparticles are biopolymer particles with a microsphere structure that can be incorporated into biodegradable polymers to control drugs and provide continuous delivery [7]. Due to their low toxicity and high biodegradability, non-antigenicity, and stability, they are actively used in targeted treatments such as for cancer, tumors, and vaccines, and they are widely used in pharmaceutical fields [11]. Albumin is a protein present in mammalian serum and is used in the manufacture of nanocapsules because of its high stability, high solubility, and excellent ease of use as a nutritional carrier. Among them, bovine serum albumin (BSA) is a protein that is nontoxic and biodegradable and thus may be easily produced as nanoparticles. BSA nanoparticles are mainly made using the desolvation method [7,11]. The drug delivery method using BSA nanoparticles has shown that the duration of drug retention in the eyes can be increased, thereby increasing the drug’s effect during treatment [10]. In addition, from the results of the cell absorption study of the breast cancer cell line (MDA-MB-231), a higher fluorescence intensity was observed in the nanoparticles containing BSA than in those not containing BSA, which showed an excellent absorption rate. The cytotoxicity result of MDA-MB-23 by MTT analysis revealed that the cell viability of cells cultured with BSA nanoparticles was over 95%, indicating the nontoxicity and high biocompatibility of the transporter. The nanoparticles containing BSA showed a cell viability of less than 20%, which means that they are effective for anticancer activity and are successfully released from cancer cells to maintain their function [12]. The desolvation method has the advantage of being able to obtain nanoparticles with a simple production method and a small size. It is a method of obtaining a nano-type precipitate by causing phase separation using a solvent such as ethanol or acetone [11]. The desolvated protein particles have a high-density structure and considerable intracellular transfer potential [13]. The soak and release method involves soaking the lens in a solution, suspension, or emulsion containing drugs to store the drugs in a contact lens and has the advantage of being simple and inexpensive [14]. Vitamin A is a retinal pigment component of the eye that helps the eye function and maintain vision, and vitamin A deficiency causes vision loss, nyctalopia, ophthalmoxerosis, epithelial cells, and mucous membrane degeneration [15]. Tretinoin (ATRA) is a vitamin A derivative, is included in the retinols of retinoids, and is a major substance that regulates the growth and differentiation of epidermal cells [16]. It also has antibacterial and anti-inflammatory effects and is used in various areas such as acne treatment, ophthalmoxerosis relief, Stevens–Johnson syndrome, keratoconjunctivitis sicca, and epithelial tumor treatment [17,18]. Tseng S. C. G, Maumenee A. E et al. (1985) evaluated the clinical efficacy of various dry eye treatments using 0.01% and 0.1% (weight/weight) topical all-trans retinoic acid points. A total of 22 patients with keratoconjunctivitis sicca, Stevens–Johnson syndrome, regular pemphigoid, and surgery for radiation-induced dry eye were treated using topical all-trans retinoic acid coordination, and all patients showed clinical improvement in symptoms, vision acuity, rose Bengal staining, or the Schirmer test [18]. Therefore, we picked BSA as a nano-carrier to deliver ATRA for efficient delivery and improved therapeutic efficacy. So far, some research has been performed on drug delivery contact lenses, but little research has been performed on drug delivery systems according to the method of adding protein nanoparticles. Also, significantly fewer studies have used ATRA in hydrogel lenses. Therefore, in this study, optical and physical properties of contact lenses such as spectral transmittance, wettability, tensile strength, drug delivery systems, and antibacterial action were measured according to the method of adding protein nanoparticles. Through this, we would like to confirm its function as an ophthalmic hydrogel lens and its potential as a drug delivery system.

## 2. Materials and Methods

### 2.1. Reagents and Materials

**Figure 1:**
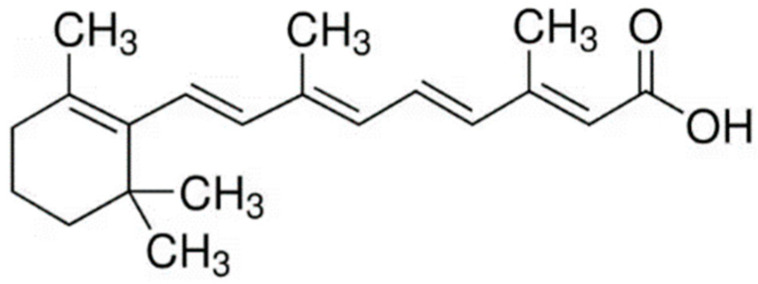
Chemical structures of Tretinoin.

2-hydroxyethyl methacrylate (HEMA, Sigma-Aldrich, St. Louis, MO, USA, assay: 97%), a cross-linker ethylene glycol dimethacrylate (EGDMA, Sigma-Aldrich, USA, assay: 98%), a thermal initiator azobisisobutyronitrile (AIBN, Junsey, Tokyo, Japan, assay: 98%), and 2-hydroxy-2-methylpropiophenone (2H2M, Sigma-Aldrich, USA, assay: 97%), which are photoinitiators, were used as additives. In addition, Tretinoin (ATRA, Sigma-Aldrich, USA, Quality Level: 300) was used as an additive, and Sigma-Aldrich products were used for all other reagents. The structural formula of the additive of the material used in the experiment is shown in Figure 1.

### 2.2. Nanoparticle Manufacturing

**Figure 2:**
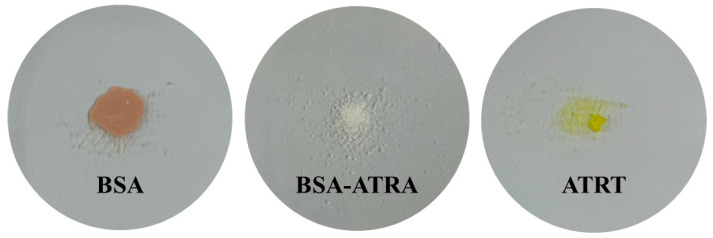
Form of manufactured nanoparticles and ATRA.

**Figure 3:**
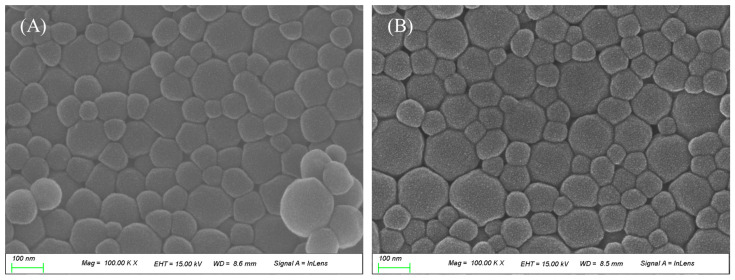
Surface analysis using SEM images [(**A**): BSA; (**B**): BSA-ATRA].

BSA nanoparticles were prepared using a previously published desolvation method [19]. In summary, 0.2 g of BSA is added to 2 mL of tertiary distilled water and stirred at 800 rpm to disperse BSA. An amount of 8 mL of ethanol is continuously added to the dispersed BSA solution at a constant rate for 1 min to induce BSA to aggregate into nanoparticles, and then 160 μL of glutaraldehyde solution (GA) is added and stirred at 800 rpm for 18 h. To separate and purify BSA nanoparticles, the supernatant is removed by centrifugation at 12,000 rpm for 15 min, and tertiary distilled water is added to the volume of the removed supernatant, followed by ultrasonication for 15 min to redisperse the aggregated BSA nanoparticles. Finally, freeze-drying is performed for 7 days to obtain nanoparticles. BSA-ATRA nanoparticles were modified with reference to F Meng, F J Liu et al. (2021) [20]. First, 0.02 g of BSA was added to 2 mL of tertiary distilled water and dissolved by ultrasonic waves. After that, 0.0016 g of ATRA was added to 8 mL of ethanol and dissolved by ultrasonic waves. The solution containing ethanol was slowly added to the solution containing distilled water. At this time, the solution was added at a ratio of 1:4 at 500 rpm using a magnetic stirrer. After addition, 50 μL of 0.25% GA was added and stirred at 500 rpm for 18 h. To separate and purify the nanoparticles, the supernatant was removed by centrifugation at 12,000 rpm for 30 min. After removing the supernatant, 1 mL of tertiary distilled water was added and redispersed. Finally, it was freeze-dried for 7 days to obtain nanoparticles. Each of the prepared nanoparticles is shown in Figure 2. SEM images of each sample are shown in Figure 3.

### 2.3. Polymerization

HEMA, EGDMA, and 2H2M were used as basic combinations, named Ref, and the samples used in the experiment were named A, B, and C, respectively. The samples were stirred using a vortex for 30 min and ultrasonic waves for 1 h, and they were photopolymerized for 1 min and 45 s using a mold casting method. Among them, A was completed by adsorbing ATRA by soaking it in a solution with ethanol after polymerization at 37 °C for 30 min. The mixing ratio for preparation is shown in Table 1.

### 2.4. Measuring Device and Methods

#### 2.4.1. Evaluation of Optical and Physical Properties of Contact Lens

The prepared hydrogel lens was hydrated in a 0.9% concentration of NaCl physiological saline for 24 h, and then its optical and physical properties were evaluated using spectral transmittance, refractive index, water content, contact angle, and tensile strength. All measurements presented in this study were repeated five or more times to increase the accuracy of the experiment. Samples were compared and analyzed as measured average values. All experiments were conducted at room temperature, and samples were stored away from direct sunlight. The samples were made in a size of 1 cm using a mold with a refractive power of 0.00D. The mixing ratio for preparation is shown in Table 1.

#### 2.4.2. Spectral Transmittance

Spectral transmittance was measured using Cary 60 UV-vis (Alient Technologies, Santa Clara, CA, USA) and was measured according to ISO 8599:1994 [21]. In addition, the ultraviolet region and the visible ray region were measured by classifying them into UV-B (280–315 nm), UV-A (320–380 nm), and Vis (380–780 nm); the transmittance for each region was measured five times each; and the average of the values expressed as percentages was calculated and determined.

#### 2.4.3. Refractive Index

The refractive index was measured using an ABBE Refractometer NARIT-2T (ATAGO, Tokyo, Japan), and it was measured based on Ophthalmic optics-Contact lenses-Part 4: Physicochemical properties of contact lens materials, 4.5. Refractive index. Moisture on the lens surface was removed using a wiper that generates less lint. The average value measured by repeating each sample five times was used.

#### 2.4.4. Water Content

Measurement of the water content was performed using an electronic scale PAG 214C (Ohaus, Parsippany, NJ, USA), and the water content was measured using a gravimetric method based on Ophthalmic optics-Contact lenses-Part 4: Physical chemical properties of contact lens materials, 4.6. water content. Water on the lens surface was removed using a dry blotting method, and the weight of the sample was measured. The weight of the dried sample was dried at 500 W for 10 min in a microwave oven, and then the weight of the dried sample was measured using an electronic scale. After measuring each weight, the water content was measured by converting it to the following equation. The water content was measured by repeatedly measuring each sample 5 times, and the average value was used.

$$W.C.^{*}\left(\%\right)=\frac{Sample^{Wet^{**}}-Sample^{Dry^{***}}}{Sample^{Wet^{**}}}\times 100$$

* W.C.: Water content;
** $Sample^{Wet}$: water-contained sample;
*** $Sample^{Dry}$: dried sample.

#### 2.4.5. Tensile Strength

Tensile strength was measured with a tensile test machine AGS-X 20N (SHIMADZU, Kyoto, Japan). When a force of 0 to 2.00 kgf was applied to both sides of the sample at a rate of 10 mm/1 min while the surface moisture of the sample was removed, the maximum damage to the lens was measured and analyzed as a tensile strength value.

#### 2.4.6. Contact Angle

The contact angle was measured using a contact angle meter Phenix-300 Touch (SEO, Incheon, Republic of Korea) and measured according to the Sessile drop method. After removing moisture from the surface of the lens using a wiper (Kim Tech) that generates less lint, distilled water was dropped on the surface of the contact lens in a flat state to measure the angle, and the converted contact angle was measured using the baseline of Man. Curve.

#### 2.4.7. Atomic Force Microscope (AFM)

The Atomic Force Microscope (AFM) XE-100 (Park Systems, Suwon, Republic of Korea) was used to analyze the surface roughness and shape of the manufactured contact lenses. The AFM is an analysis equipment that can obtain atomic or nano-level surface information on the sample surface with a fine probe, and the molecular composition of the local surface of the sample and the topographic map of the lens surface were measured.

#### 2.4.8. Drug Concentration

Drug concentrations were measured using a Carey 60 UV-vis (Alient Technologo-gies, USA). Each A, B, and C sample was used by immersing in a 10 mL phosphate-buffered saline (PBS) solution at room temperature. The experiment was conducted by holding the base lines with a PBS solution, the concentration of eluting drug was measured over time, and the absorbance value was checked at 350 nm, which is the maximum absorption wavelength of ATRA. The absorption coefficient of ATRA was calculated using ε_350_ = 48,800 M^−1^cm^−1^ at 350 nm, and the drug elution concentration was determined by the following equation.

$$\mu M^{*}=\frac{A^{***}}{\epsilon b^{**}}$$

* μM: drug elution concentration;
** ϵb: molar absorptivity × path length;
*** *A*: absorbance.

#### 2.4.9. Antimicrobial Property

In order to evaluate the antimicrobial activity against *Staphylococcus aureus*, the analysis was performed using a dry film, 3M Petrifilm^TM^ (Sigma-Aldrich, USA). After 1 mL of the sample solution was injected into the dried medium, the solution was cultured in a thermostat at 35 °C ± 1 °C for about 24 h, and the antimicrobial activity against *Staphylococcus aureus* and *Escherichia coli* was confirmed.

## 3. Results and Discussion

### 3.1. Spectral Transmittance

**Figure 4:**
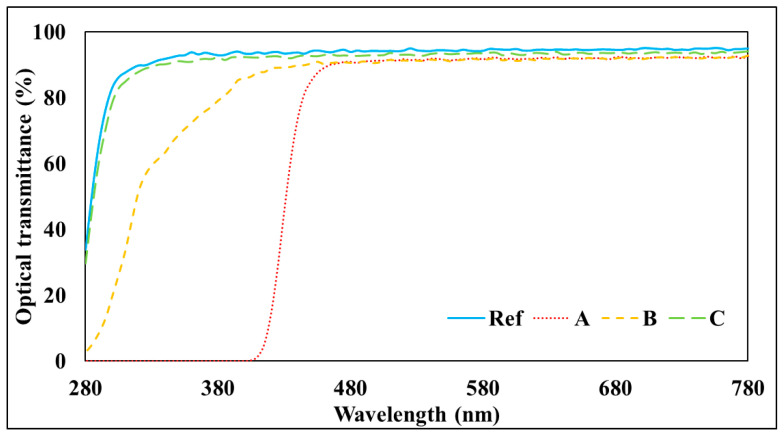
The effect of spectral transmittances of samples.

The spectral transmittance was measured by classifying it into an ultraviolet region including UV-B (280–315 nm) and a visible ray region including VIS (380–780 nm). As a result of measuring the spectral transmittance of the prepared hydrogel lens, it was confirmed that in the case of the visible ray region, 88% or more of all samples were found, thereby satisfying the basic contact lens requirements of ANSI Z80.20:2004 [22]. In the case of the ultraviolet region, it was confirmed that ultraviolet rays were blocked in A and B, but not C. In particular, A showed excellent blocking rates in both UV-B and UV-A regions. Therefore, it is determined that ATRA can be used as a contact lens material for UV protection. However, in the case of C, since albumin nanoparticles containing ATRA were prepared, it is determined that the unique ultraviolet blocking characteristics of ATRA disappeared, and thus ultraviolet rays could not be blocked. The graph of the spectral transmittance of each sample is shown in Figure 4.

### 3.2. Refractive Index and Water Content

**Figure 5:**
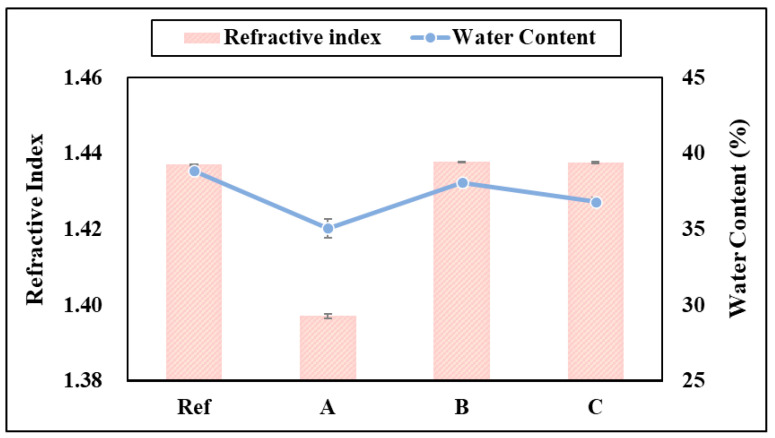
The effect of the refractive index and water content of samples.

As a result of measuring the refractive index of the prepared hydrogel lens, it was found to be 1.4370 for Ref, 1.3971 for A, 1.4378 for B, and 1.4376 for C. Accordingly, it was confirmed that all samples except A, which was the lowest, showed no significant change in refractive index. In addition, as a result of the water content measurement, it was found to be 38.83% for Ref, 35.06% for A, 38.07% for B, and 36.80% for C. Therefore, it was confirmed that the water content was lower than that of Ref in all samples. This was determined to be the result of the decrease in the water content of the photopolymerization lens with ATRA added. A previous study was conducted on the physical properties of the ATRA-added contact lens according to thermal polymerization and photopolymerization. As a result, it was confirmed that in the case of photopolymerized samples, the water content decreased to 38.26~37.70% as the ratio of ATRA added increased. A graph of the refractive index and water content of each sample is shown in Figure 5.

### 3.3. Contact Angle and AFM

**Figure 6:**
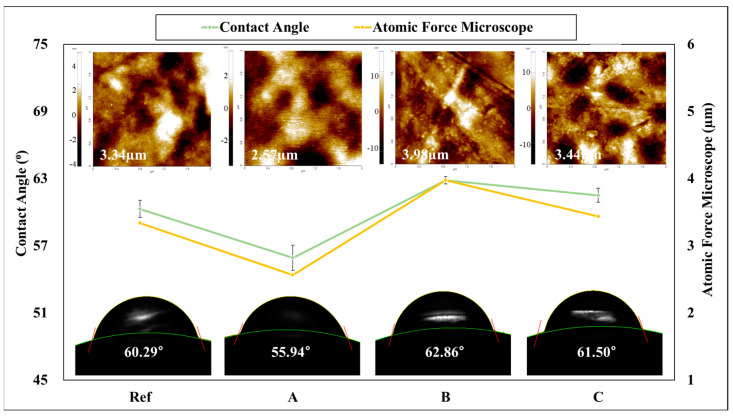
The effect of the contact angle and AFM images of samples.

As a result of measuring the contact angle and AFM of the hydrogel lens manufactured for wettability and roughness evaluation, the contact angle of the Ref was measured to be 60.29°, and the contact angle of A was 55.94°, that of B was 62.86°, and that of C was 61.50°. In addition, the average surface roughness value (Ra) of the lens was measured to be Ref 3.34 μm, that of A was 2.57 μm, that of B was 3.98 μm, and that of C was 3.44 μm. Accordingly, it was confirmed that A has high wettability and has excellent wearability. The contact angle is an angle at which liquid and gas are thermodynamically balanced on a solid surface, and if the contact angle is low, the surface energy is high, so it is determined to be highly wettable. In addition, it was confirmed that the result of the contact angle and AFM was highest in B and lowest in A. It is determined that the result of a similar pattern appeared because the surface roughness of the lens affects the contact angle [23]. The AFM and contact angle images and graphs of each sample are shown in Figure 6.

### 3.4. Tensile Strength

**Figure 7:**
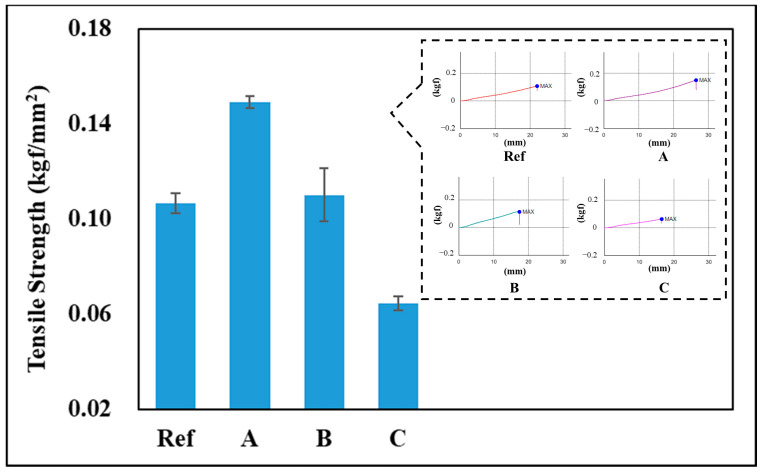
The effect of the tensile strength graph of samples.

As a result of measuring the tensile strength of the hydrogel lens prepared for durability evaluation, the tensile strength of Ref was 0.107 kgf/mm^2^, that of A was 0.1491 kgf/mm^2^, that of B was 0.1102 kgf/mm^2^, and that of C was 0.0644 kgf/mm^2^. Therefore, it was confirmed that the tensile strength of A and B, excluding C, was higher than that of Ref. In the case of C, it is determined that albumin nanoparticles containing ATRA became new particles and exhibited different characteristics. In contrast, A and B contain BSA. Therefore, it is determined that the mechanical properties improve due to the properties making the material of BSA more viscoelastic or harder [24]. Among them, it is determined that the network of the surface coating layer due to the soak of A is attached to the three-dimensional polymer network of the hydrogel contact lens to form a multiple structure, thereby improving mechanical properties compared to B [25]. A graph of the tensile strength of each sample is shown in Figure 7.

### 3.5. Drug Delivery

**Figure 8:**
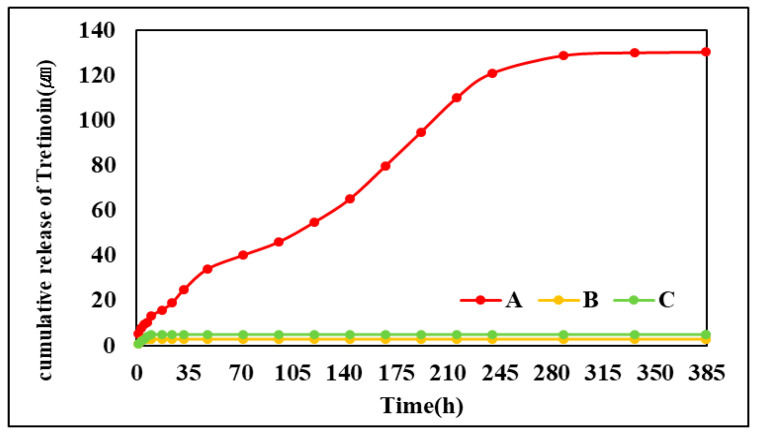
Concentration of drug cumulative release of samples.

As a result of measuring the release concentration and release duration of ATRA, A released 130.35 µm over 336 h, and very small amounts of B and C were released. Accordingly, A could be released continuously for a long time, resulting in excellent results, but it was confirmed that B and C could not be released and thus could not deliver the drug. The drug delivery system using the contact lens reduces the loss of drugs due to tears, but it is difficult to continuously release a large amount in the early stages, so various studies are being conducted to solve this problem [26]. Therefore, the hydrogel contact lens using both the albumin addition and soak and release methods studied in this paper can control the duration and effect of the drug and are considered suitable as a material for a carrier to deliver the drug. The graph of drug delivery is shown in Figure 8.

### 3.6. Antibacterial Properties

**Figure 9:**
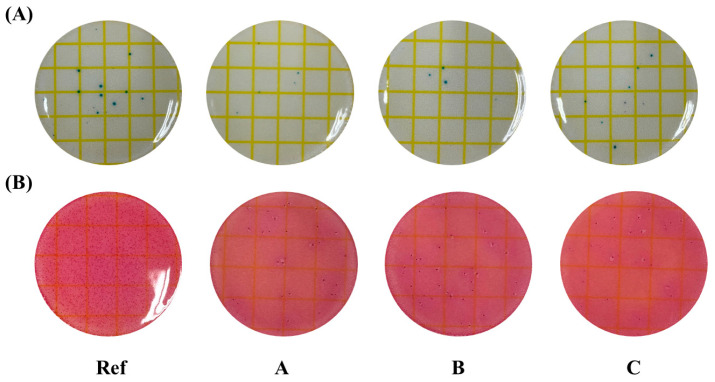
Antimicrobial properties of the samples [(**A**): *Staphylococcus aureus;* (**B**): *Escherichia coli*].

For the antibacterial test, the antibacterial activity against microorganisms was confirmed using a dry film method. The antimicrobial property of the contact lens with ATRA against *Staphylococcus aureus* and E. coli was confirmed. *Escherichia coli*, fungus, and *Staphylococcus aureus* are common microorganisms that cause ocular diseases, including keratohelcosis and conjunctivitis [27]. As a result of the measurement, the Ref to which ATRA was not added showed somewhat more of both *Staphylococcus aureus* and *Escherichia coli*. On the other hand, it was confirmed that all groups to which ATRA was added had excellent antimicrobial properties because there were few microorganisms. The measurement results of antimicrobial activity are shown in Figure 9.

### 3.7. Limitations

In this study, it was confirmed that the drug was released only in Sample A. Therefore, it was confirmed that the method of adding an additive greatly affects the drug release regardless of the presence or absence of nanoparticles. In addition, it is an opinion that protein nanoparticles are key to drug release and are limited in confirming their utility. In the future, it is thought that experiments on the above conditions are necessary.

## 4. Conclusions

In this study, BSA nanoparticles and BSA-ATRA nanoparticles were manufactured, and nanoparticles and ATRA were added to a hydrogel contact lens in three ways to compare and analyze the physical properties of each method. As a result of analyzing the physical properties of the manufactured contact lens, antibacterial results were excellent in all groups, and all physical properties except for this each showed different characteristics. In particular, A showed the best UV protection, wettability, tensile strength, and drug delivery duration, and it was confirmed that B and C could not deliver the drug, due to the release of a very small amount of the drug. Accordingly, by analyzing the physical properties of the contact lens according to the three methods, it is concluded that a system that is highly useful as various ophthalmic lenses can be constructed. In addition, it suggests that if the optimal drug delivery system according to the addition method of ATRA and BSA is properly utilized for a contact lens, a lens with various functions such as UV protection, wettability, durability, and antibacterial properties will be created.

## Figures and Tables

**Table 1:** Compositions of hydrogel lens samples (unit: %).

Sample	HEMA	EGDMA	2H2M	ATRA	BSA	BSA-ATRA
Ref	98.52	0.99	0.49	-	-	-
A	97.54	0.98	0.49		0.99	
B	97.06	0.97	0.49	0.49	0.99	
C	97.54	0.98	0.49			0.99

## Data Availability

The original contributions presented in this study are included in the article. Further inquiries can be directed to the corresponding author.
